# Comparison of first-line chemotherapy based on irinotecan or other drugs to treat non-small cell lung cancer in stage IIIB/IV: a systematic review and meta-analysis

**DOI:** 10.1186/s12885-015-1978-2

**Published:** 2015-12-16

**Authors:** Xue-Qin Yang, Chong-Yi Li, Ming-Fang Xu, Hong Zhao, Dong Wang

**Affiliations:** Cancer Center, Daping Hospital, Third Military Medical University, No.10 Changjiang, Daping Yuzhong District, Chongqing 400042 China; Department of Medical Protection, 537 Hospital of the Chinese People’s Liberation Army, Baoji, 721006 China

**Keywords:** Irinotecan, Chemotherapy, Non-small cell lung cancer, Meta-analysis

## Abstract

**Background:**

To compare the efficacy and toxicity of irinotecan-based chemotherapy (IBC) and non-irinotecan-based chemotherapy (NIBC) as first-line treatment for stage IIIB/IV non-small cell lung cancer (NSCLC).

**Methods:**

PubMed, EMBASE, the Cochrane Central Register of Controlled Trials (CENTRAL), abstracts from the annual meetings of ASCO and the ESMO up to 2014 were searched for randomized controlled trials (RCTs) that compared IBC with NIBC. Data on overall survival (OS) and progression-free survival (PFS) were meta-analyzed to provide hazard ratios (HRs), while data on overall response rate (ORR) and frequencies of toxicity were meta-analyzed to provide relative risk ratios (RR).

**Results:**

Seven RCTs (6 RCTs from Asian population and 1 from non-Asian population) involving 1473 patients with previously untreated stage IIIB/IV NSCLC were included in the meta-analysis. IBC and NIBC were associated with similar ORR (RR: 1.08, 95 %CI: 0.94 to 1.23, *p* = 0.30), OS (HR: 0.97, 95 %CI: 0.88 to 1.07, *p* = 0.56), and PFS (HR: 1.02, 95 %CI: 0.97 to 1.08, *p* = 0.38). However, the subgroups between Asian and non-Asian patients differed significantly in OS (HR: 0.94 vs 1.87, *p* = 0.007). There was no significant difference for hematological toxicity (RR: 0.79, 95 %CI: 0.60 to 1.04, *p* = 0.09) and significant worse for non-hematological toxicity (RR: 2.28, 95 %CI: 1.60 to3.24, *p* < 0.001), when IBC compared to NIBC.

**Conclusions:**

As the available evidence suggests that IBC and NIBC are equivalent in terms of ORR, PFS, OS, at least in Asian patients, we recommend that IBC be considered as a first-line treatment in Asian patients with stage IIIB/IV NSCLC. However, the non-hematological toxicity of IBC must be considered.

**Electronic supplementary material:**

The online version of this article (doi:10.1186/s12885-015-1978-2) contains supplementary material, which is available to authorized users.

## Background

Lung cancer is the leading cause of cancer-related deaths worldwide, and approximately 80 % of patients with lung cancer are non-small cell lung cancer (NSCLC). Two-thirds of patients with NSCLC are diagnosed when they are already in stage IIIB or IV [1–3]. Traditionally, chemotherapy is the first choice for the treatment of this status. Currently, however, the mutation status of the epidermal growth factor receptor (EGFR) and the arrangement status of anaplastic lymph kinase (ALK) are independent pathologic types in NSCLC. EGFR tyrosine kinase inhibitors (EGFR-TKIs) and ALK inhibitors show promise for treating these types and are recommended as the first choice by National Comprehensive Cancer Network (NCCN). However, the rate of EGFR positive mutation is about 20 % and ALK arrangement rate is only about 5-7 % [4]. Therefore chemotherapy is still recommended as the 1st-line treatment for stage IV NSCLC patients without EGFR mutation, ALK fusion gene arrangement or unknown for these gene mutation statuses in the NCCN guideline. Guidelines of NCCN recommend first-line treatment with platinum-doublet chemotherapy, which include paclitaxel, docetaxel, gemcitabine, etoposide, vinblastine, vinorebine, pemetrexed, and albumin-bound paclitaxel [5].

Chemotherapy based on the topoisomerase I inhibitor irinotecan may provide another option for first-line treatment of advanced NSCLC. Irinotecan-based chemotherapy (IBC) has already been shown to be effective against colorectal cancer (CRC), lung cancer, gastric cancer and gynecologic neoplasms [6–9]. NCCN guidelines recommend IBC as first-line treatment of metastatic CRC and extensive-stage small-cell lung cancer (SCLC). Several studies, primarily in Asian patients, suggest that IBC and non-irinotecan-based chemotherapy (NIBC) lead to similar clinical benefit and different toxicity profiles when used to treat stage IIIB or IV NSCLC. However, NCCN guidelines do not currently recommend IBC as first-line therapy for NSCLC.

Therefore we performed a systematic review and meta-analysis of randomized controlled trials comparing IBC and NIBC in patients with stage IIIB or IV NSCLC. The goal was to assess the current evidence on the efficacy and safety of IBC in a relatively large cohort in order to help clinicians identify whether IBC is appropriate as first-line chemotherapy regimen.

## Methods

### Search strategy

We searched PubMed, EMBASE and the Cochrane Central Register of Controlled Trials (CENTRAL) up to April 2014 without any limitations on publication year or language. The search terms “non-small cell lung cancer”, “NSCLC”, “irinotecan”, and “CPT-11”were used. Abstracts published at the annual meetings of the American Society of Clinical Oncology and the European Society for Medical Oncology from 2000 onwards was also searched. Reference lists in original articles and review articles were manually searched to identify additional relevant trials.

### Study selection

Studies were included if they (1) involved patients previously untreated with chemotherapy, locally advanced (stage IIIB) or metastatic (stage IV) NSCLC; (2) were randomized controlled trials (RCTs) with an IBC arm and NIBC arm; (3) were published in full as original articles or as abstracts with sufficient detail, as long as the first author of the study was able to confirm the full results.

### Data extraction

All data were extracted independently by two investigators (XQY and CYL) using a standardized form. Extracted data included first author, publication year, patient enrollment period, ethnicity, median age, total number of patients, number of patients eligible for response evaluation, performance status, chemotherapy regimens, hazard ratios (HRs) for progression-free survival (PFS) and overall survival (OS) and the associated 95 % confidence intervals (CIs), rates of treatment-related deaths and rates of grade 3–4 toxicity effects, such as anemia, neutropenia, thrombocytopenia, nausea/vomiting, diarrhea, and treatment-related death. Disagreements were resolved by discussion with a third author (MFX).

If HR was not directly reported, the log HR and variance were estimated using the method of Tierney et al. [10]. The HRs of OS and PFS were extracted directly from the text, calculated from the reported number of events and the corresponding log-rank *p* value, or read off the survival curves.

### Study quality assessment

We assessed the methodological quality of the studies using the modified Jadad score [11], which evaluates whether the trial responds adequately to the following four questions: (1) whether an appropriate randomization method is reported (0–2 points); (2) whether randomization concealment is reported (0–2 points); (3) whether an appropriate blinding method is reported (0–2 points); and (4) whether withdrawals and dropouts are reported (0–1 points).

### Statistical analysis

All meta-analyses were performed using Review Manager 5.2 (Cochrane Collaboration) except publication bias was calculated using STATA SE 12.1 package (StataCorp, College Station, TX); other statistical tests were performed using SPSS for Windows 13.0 (IBM, Chicago, IL). PFS and OS for IBC and NIBC patients were compared using HRs, while dichotomous data for the two treatment groups were compared using risk ratios (RRs). The comparisons were carried out such that RR >1 indicated higher overall response or toxicity in the IBC group, and HR >1 indicated more deaths or progression in the IBC group. RR, OS and PFS were also performed further subgroup analyses between Asian and non-Asian patients. Statistical heterogeneity among trials was assessed using the chi-squared test and expressed using the I^2^ index [12]. Normally the fixed-effects model was used and weighted according to the Mantel-Haenszel method. When pooled data showed considerable heterogeneity (*p* < 0.1 or I^2^ > 50 %), a random-effects meta-analysis model was used. All *p* values were two-sided, with *p* < 0.05 defined as the threshold of statistical significance.

## Results

### Trial flow for meta-analysis

**Fig. 1:**
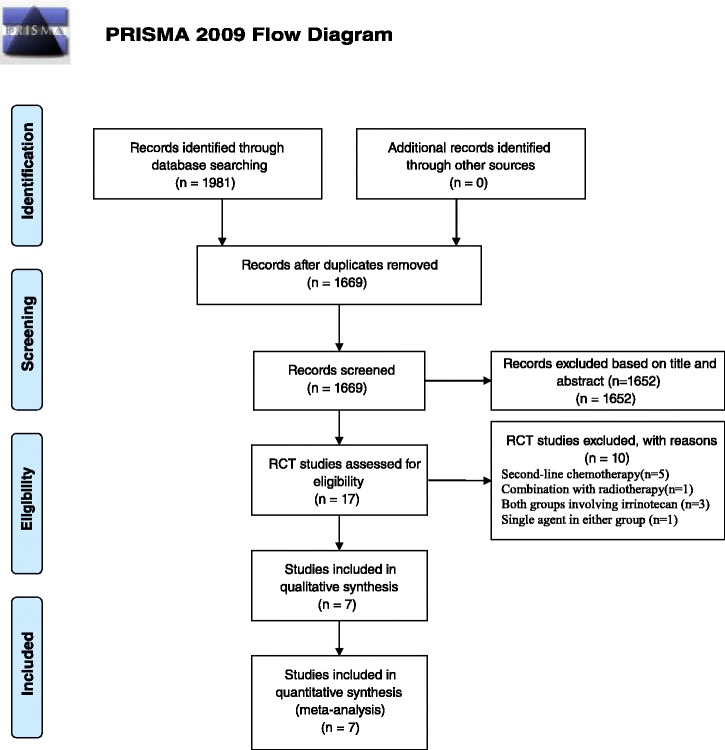
PRISMA flowchart

Database searches turned up 1981 potentially eligible publications, of which 312 were excluded as doublet report and 1652 were excluded based on title and abstract review. Of the remaining 17 publications, 6 were excluded because they were RCTs that employed IBC and NIBC as second-line chemotherapy or combined with radiation, four were excluded as single agent in either group or both groups involved irinotecan. In the end, six full papers and one abstract involving 1473 patients with stage IIIB/IV NSCLC were included [13–19] (PRISMA flowchart see Fig. 1).

### Characteristics of included studies

We identified seven RCTs meeting the inclusion criteria: three Phase III RCTs [13, 15, 18], Four Phase II RCTs (Table 1 and Additional file 1) [14, 16, 17, 19]. Four trials involved patient cohorts in Japan [13–15, 18], and one trial each was from China [17] and South Korea [16]. Only one trial was from a non-Asian population, namely North Americans [19].

**Table 1:** Summary of studies

Author	Phase	Race	Jadad score	Group	Regimen	N	Eligible for evaluation	Male (%)	Median age	PS 0-1(%)	Stage IV(%)	Histology Ad. (%)	Median cycles
Negoro S et al.[13] 2003	III	Japanese	5	IP	P: 80mg/m^2^ day 1, I: 60mg/m^2^, day 1, 8, 15, q 28days	133	129	76	64	94	62	60	3
				PV	P: 80mg/m^2^ d1, V: 3mg/m^2^, day 1, 8, 15, q 28days	133	122	80	64	94	63	60	2
Yamamoto N et al.[14] 2004	II	Japanese	5	DI	D: 60mg/m^2^ day 8, I: 60mg/m^2^, day 1, 8, q 21days	57	57	67	60	100	75	77	2
				DP	P: 80mg/m^2^ day 1, D: 60mg/m^2^, day 1, q 21days	51	51	73	62	100	78	71	2
Ohe Y et al.[15] 2007	III	Japanese	5	IP	P: 80mg/m^2^ day 1, I: 60mg/m^2^, day 1, 8, 15, q 28days	151	145	67	62	100	79	83	3
				TC	T: 200mg/m^2^ day 1, C: AUC 6, day 1, q 21days	150	145	68	63	100	81	72	3
				GP	P: 80mg/m^2^ day 1, G: 1000mg/m^2^, day 1, 8, q 21days	151	146	70	61	100	79	74	3
				NP	P: 80mg/m^2^ day 1, N :20mg/m^2^ , day 1, 8, q 21days	150	145	70	61	100	82	75	3
Han JY et al.[16] 2008	II	Korean	5	IP	P: 30mg/m^2^, I :65mg/m^2^, day 1, 8, q21days	75	72	77	58	89	87	71	5
				GN	G: 900mg/m^2^, N :25mg/m^2^ , day 1, 8, q 21days	71	71	82	60	92	87	69	4
Zhao WY et al.[17] 2012	II	Chinese	4	IP	P: 25mg/m^2^ day 1–3, I :100mg/m^2^, day 1, 8, q 21days	31	31	68	59*	68	65	61	3
				GP	P: 25mg/m^2^ day 1–3, G :1000mg/m^2^, day 1, 8, q 21days	32	32	72	60*	75	59	56	3
Takiguchi Y et al.[18] 2000	III	Japanese	3	IP	P: 80mg/m^2^ day 1, I: 60mg/m^2^ , day 1, 8, 15, q 28days	104	98	75	62	94	59	NR	NR
				PV	P: 80mg/m^2^ day 1, V: 3mg/m^2^ , day 1, 8, 15, q 28days	106	101						
Rocha Lima CM et al.[19] 2004	II	American	5	GI	G :1000 mg/ m^2^ day 1, 8, I: 100 mg/m^2^, day 1, q 21 days	39	36	72	63	100	77	46	4
				GD	G: 1000mg/m^2^ day 1, 8, D: 40mg/m^2^, day 1,8, q 21days	39	36	46	57	100	79	59	4

*I* Irinotecan, *P* cisplatin, *V* vindesine, *G* gemcitabine, *N* navelbine, *D* docetaxel, *T* paclitaxel, *C* carboplatin, *NR* not reported, *Ad*. adenocarcinoma

* This is mean data, not median data.

In total, 590 patients with stage IIIB/IV NSCLC were randomized to receive IBC, and 883 patients to receive NIBC. The IBC regimen was irinotecan and platinum in five trials [13, 15–18] and irinotecan and docetaxel [14] or gemcitabine [19] in the remaining trials. None of the patients had been treated prior to trial enrollment, with the exception of six (7.7 %) patients (two in IBC and four in NIBC group) in the study of Rocha Lima et al. [19]; these six patients had recurrent/progressive disease after surgery and/or radiation therapy with no chemotherapy previously.

The trial by Takiguchi et al. [18] did not report the baseline characteristics of the two arms in its abstract, although the authors did report that patients were well-balanced with regard to age, sex, stage, and performance status (PS).

The quality of the seven trials was assessed using the modified Jadad score (Table 1). The full score was seven points. As none of the trials was double-blinded, no trials received the highest possible score.

### Overall response rate

**Fig. 2:**
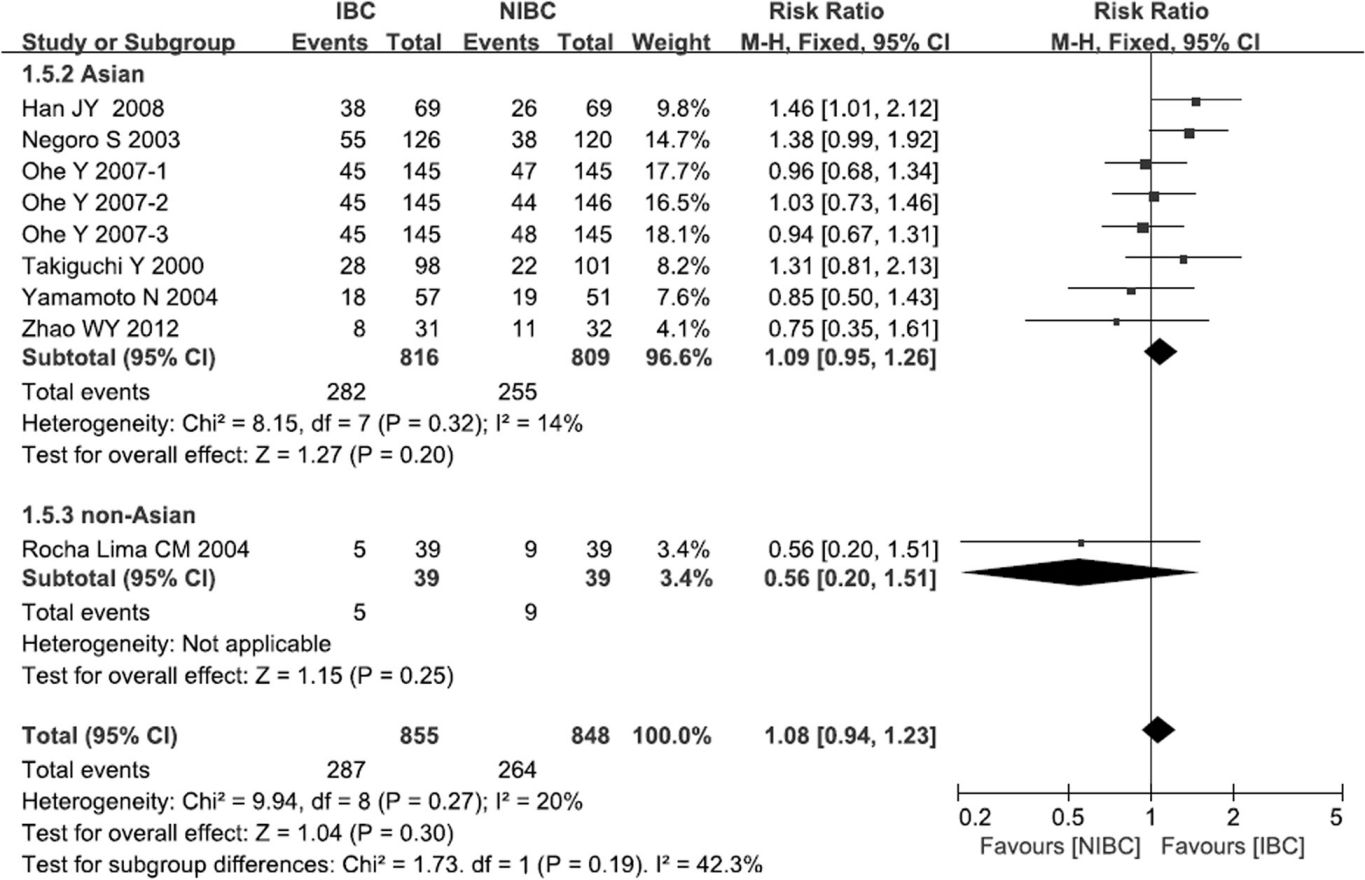
Comparison of overall response rate between irinotecan-based chemotherapy and non-irinotecan-based chemotherapy as first-line treatment in patients with stage IIIB/IV NSCLC

All seven trials in the meta-analysis reported overall response rate (ORR). Meta analysis showed that IBC group had the similar ORR compared with NIBC group (RR: 1.08, 95 %CI: 0.94–1.23, *p* = 0.30; Fig. 2). This meta-analysis was performed using the fixed-effects model since the pooled results showed no significant heterogeneity (*χ*^2^ = 9.94, *p* = 0.27; I^2^ = 20 %).

Among the seven trials, one trial reported that IBC was associated with a significantly higher ORR than NIBC (*p* = 0.041) [16], while another trial reported a tendency towards higher response rate for IBC (*p* = 0.053) [13], and the remaining trials on Asian cohorts reported similar response rates for the two treatments [14, 15, 17, 18], However, the one trial on a non-Asian population reported a tendency towards lower response rate for IBC (IBC vs NIBC: 12.8 % vs 23.1 %, *p* = 0.238) [19]. Thus we performed separate meta-analyses for Asian and non-Asian populations. The results showed that the non-Asian population had a tendency towards lower RR value compared to Asian population (RR: 0.56 vs 1.09, *p* = 0.19).

### Overall survival

**Fig. 3:**
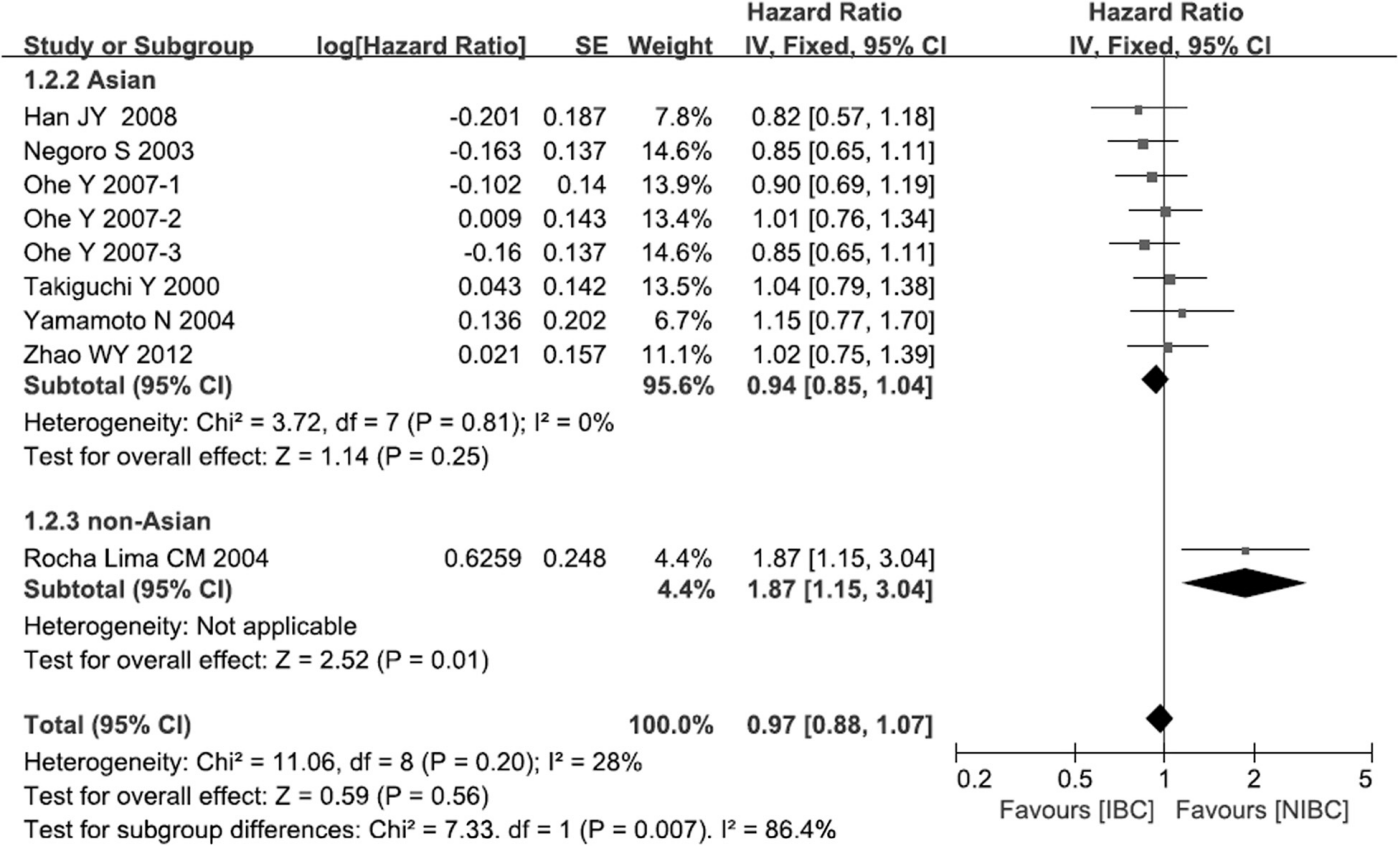
Comparison of overall survival between irinotecan-based chemotherapy and non-irinotecan-based chemotherapy as first-line treatment in patients with stage IIIB/IV NSCLC

The meta-analysis from six trials involving Asian patients reported similar OS for IBC and NIBC (HR: 0.94, 95 %CI: 0.85 to 1.04, *p* = 0.25; Fig. 3) [13–18]. However, results for the non-Asian patients showed that IBC had shorter OS than NIBC (HR: 1.87, 95 %CI: 1.15 to 3.04, *p* = 0.01; Fig. 3) [19]. These two subgroups differed significantly in OS (*χ*^2^ = 7.33, *p* = 0.007; I^2^ = 86.4 %). Overall meta-analysis of all seven trials showed similar results (HR: 0.97, 95 %CI 0.88 to 1.07, *p* = 0.56; Fig. 3).

### Progression-free survival

**Fig. 4:**
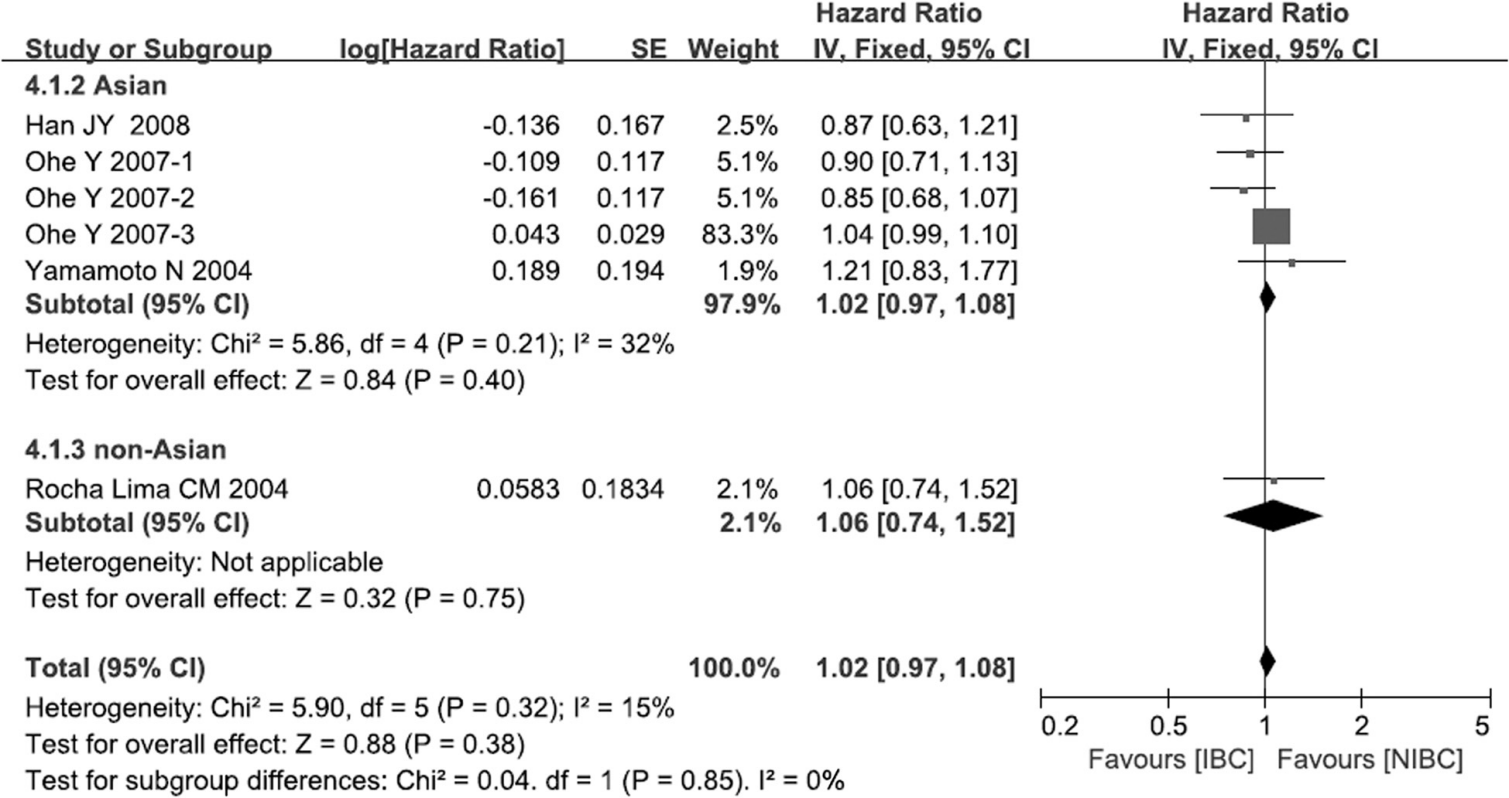
Comparison of progression-free survival between irinotecan-based chemotherapy and non-irinotecan-based chemotherapy as first-line treatment in patients with stage IIIB/IV NSCLC

Four trials involving 934 patients provided sufficient data to extract HRs for PFS [14–16, 19]. The pooled HR from four trials showed similar PFS for IBC and NIBC (HR 1.02, 95 %CI 0.97 to 1.08, p = 0.38; Fig. 4). The two subgroups did not show significant differences in PFS (*χ*^2^ = 0.04, *p* = 0.85; I^2^ = 0 %). Another two trials reported that PFS was similar for IBC and NIBC, but they did not report survival curves or log rank p-value, preventing us from including them in the meta-analysis [13, 17].

### Adverse events

#### Hematological toxicity

**Fig. 5:**
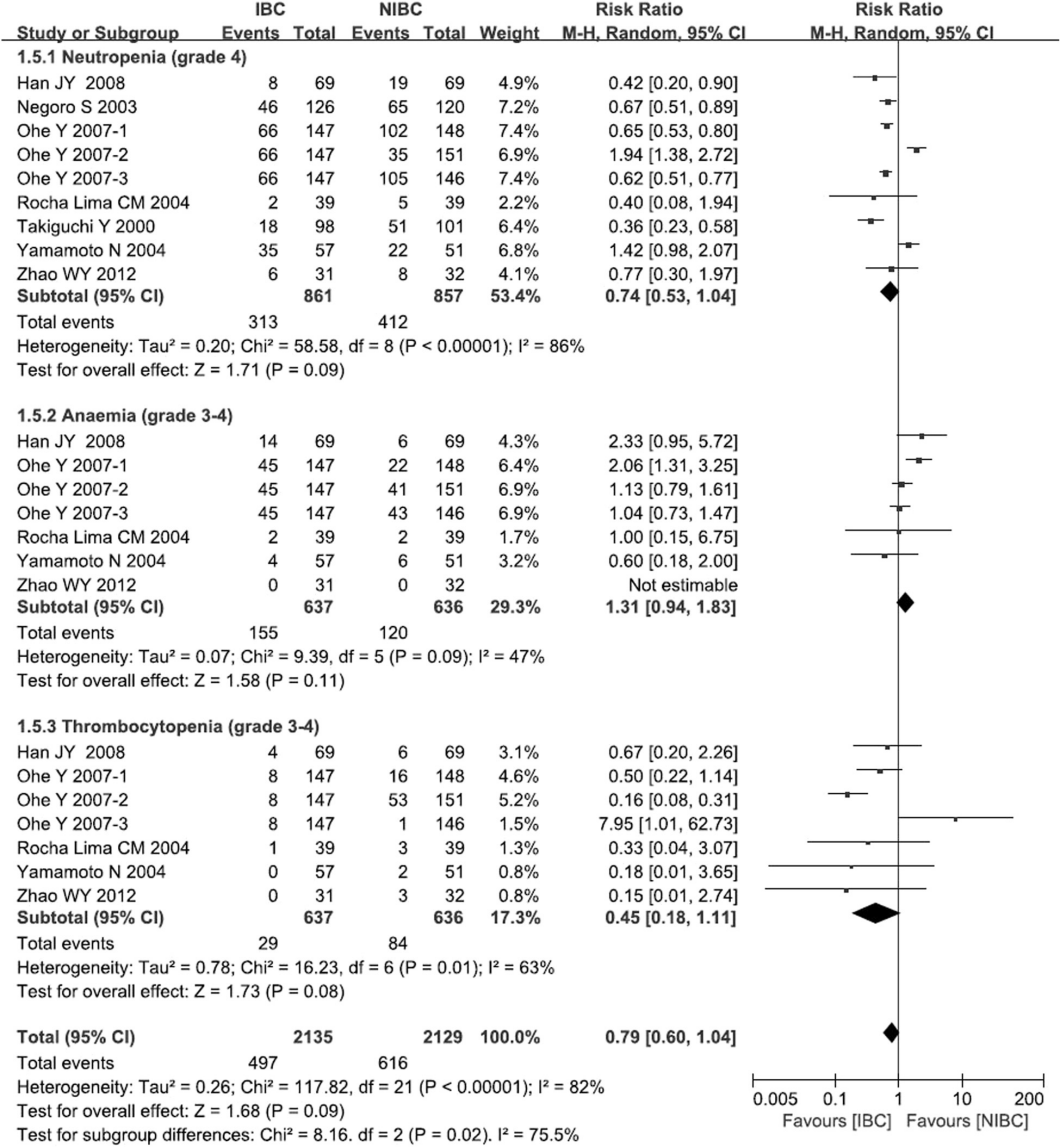
Comparison of hematological adverse events between irinotecan-based chemotherapy and non-irinotecan-based chemotherapy as first-line treatment in patients with stage IIIB/IV NSCLC

Five trials reported data on grade 3–4 hematological toxic effects, such as thrombocytopenia and anemia [14–17, 19], while all trials reported data on grade 4 neutropenia. Meta-analysis of pooled data showed that IBC and NIBC were associated with similar incidence of anemia (RR: 1.31, 95 %CI: 0.94 to 1.83, *p* = 0.11; Fig. 5), neutropenia (RR: 0.74, 95 %CI: 0.53 to 1.04, *p* = 0.09; Fig. 5) and thrombocytopenia (RR: 0.45, 95 %CI: 0.18 to 1.11, *p* = 0.08; Fig. 5) for IBC group. The overall effect for hematological toxicity also had no significant difference between IBC and NIBC (RR: 0.79, 95 %CI: 0.60 to 1.04, *p* = 0.09; Fig. 5). Pooled data for neutropenia and thrombocytopenia showed significant heterogeneity, probably because of the diverse treatment regimes used in the various trials. Therefore we used a random-effects model to perform these meta-analyses.

#### Non-hematological toxicity

**Fig. 6:**
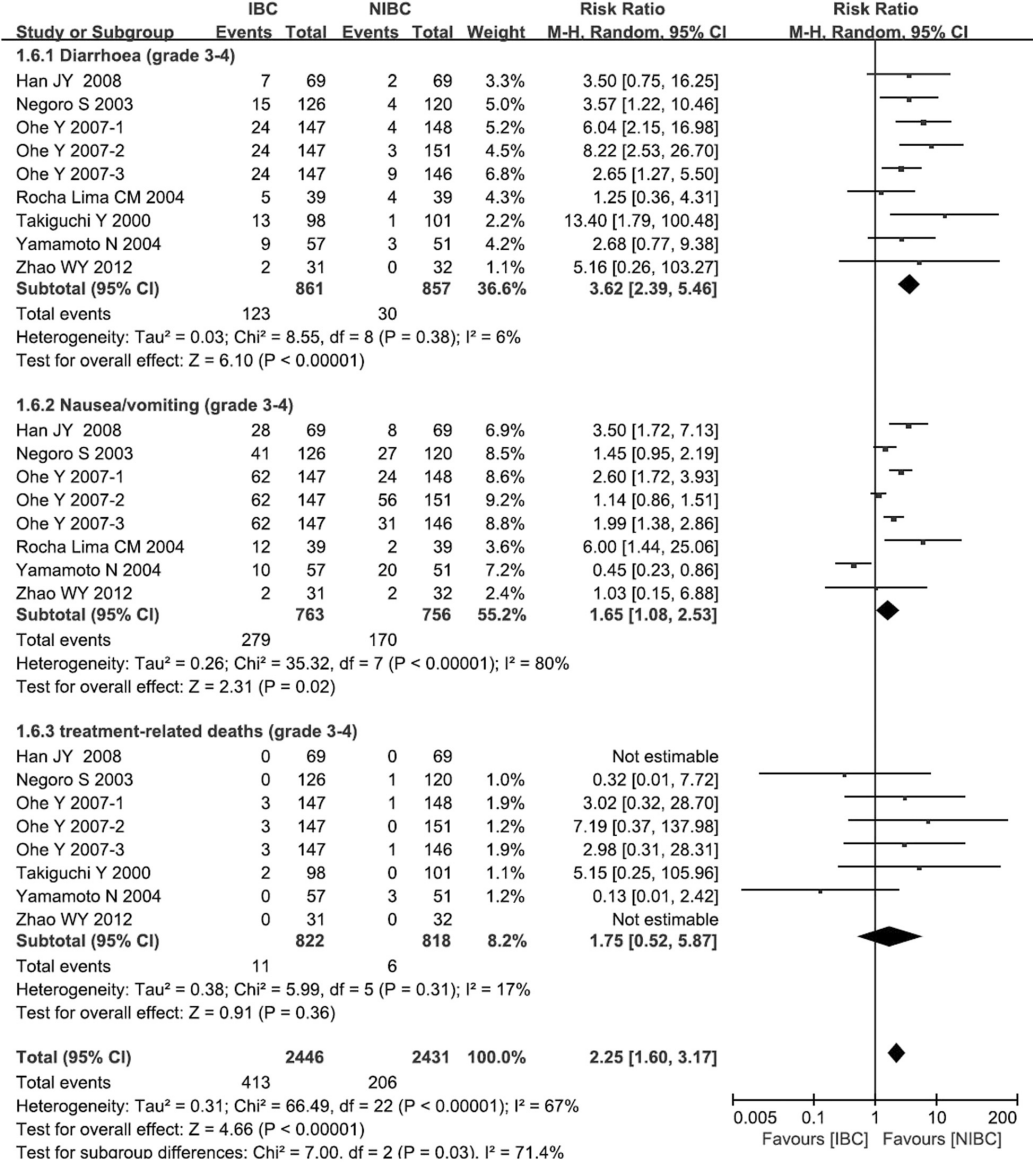
Comparison of non-hematological adverse events between irinotecan-based chemotherapy and non-irinotecan-based chemotherapy as first-line treatment in patients with stage IIIB/IV NSCLC

All seven trials reported data on grade 3–4 diarrhea. Only one trial did not report data on grade 3–4 nausea/vomiting [18], while another trial did not report data on treatment-related deaths [19]. Meta-analysis showed that IBC was associated with a higher risk of grade 3–4 diarrhea than NIBC (RR: 3.62, 95 %CI: 2.39 to 5.46, *p* < 0.001), as well as higher risk of nausea/vomiting (RR: 1.65, 95 %CI: 1.08 to2.53, *p* = 0.02; Fig. 6). In contrast, the two treatments showed similar rates of treatment-related deaths (RR: 1.75, 95 %CI: 0.52 to 5.87, *p* = 0.36; Fig. 6). The overall effect of non-hematological toxicity for IBC is worse than that of NIBC (RR: 2.25, 95 %CI: 1.60 to 3.17, *p* < 0.001; Fig. 6). Pooled data for diarrhea and treatment-related deaths showed no significant heterogeneity, but pooled data for nausea/vomiting did. Therefore all these meta-analyses were carried out using a random-effects model.

### Publication bias

**Fig. 7:**
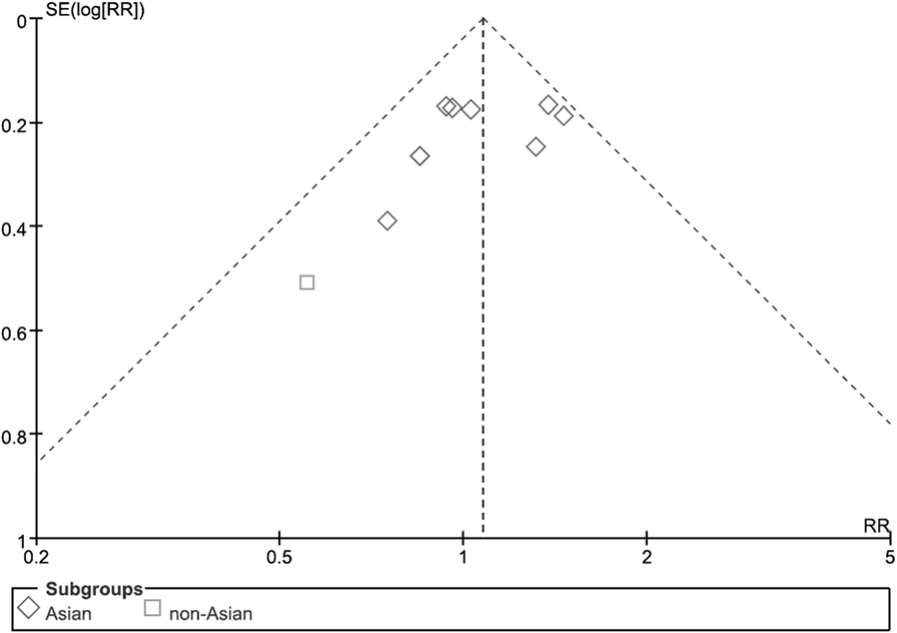
Funnel plot to assess risk of publication bias in included studies

We created a funnel plot of the included study data to assess the risk of publication bias. The plot showed no apparent bias (Egger’s test: *p* = 0.177) (Fig. 7).

## Discussion

The results of this meta-analysis showed that the two types of chemotherapy were associated with similar overall treatment response rate and OS in NSCLC patients with stage IIIB or IV. Also, these two regimens have similar PFS, however, which may not be very convincing as the PFS data of this study is only from three Asian trials and one non-Asian trial. There was no significant difference for hematological toxicity (RR: 0.79, 95 %CI: 0.60 to 1.04, *p* = 0.09) and significant worse for non-hematological toxicity (RR: 2.28, 95 %CI: 1.60 to3.24, *p* < 0.001), when IBC compared to NIBC. As chemotherapy is recommended as the 1st-line treatment in the patients with stage IV NSCLC without EGFR mutation, ALK fusion gene arrangement or unknown for these gene mutation statuses in NCCN guideline, we recommend that IBC be considered a 1st-line treatment for NSCLC patients with stage IIIB or IV, especially for the patients without above mutation or gene arrangement. However, the non-hematological toxicity of IBC must be considered. As irinotecan-induced toxicity, including neutropenia and diarrhea, is associated with a single-nucleotide polymorphism (SNP) in the UTG1A1 gene [20] and such that genetic testing for this SNP was approved in 2005 by the US Food and Drug Administration (FDA) as a screening method to identify patients at higher risk of adverse events with IBC, testing for this SNP may help guide proper choice of first-line chemotherapy against NSCLC.

This meta-analysis included 6 Asian trials and only 1 non-Asian trial. The meta-analysis result for OS differed significantly between non-Asians and Asians (HR: 1.87 vs 0.94, *p* = 0.007), and for ORR had a tendency towards lower RR value when non-Asians compared to Asian population (RR: 0.56 vs 1.09, *p* = 0.19). However, one non-Asian trial with a small sample study may come to a detrimental result for irinotecan in a non-Asian population, which raise the question of whether our meta-analysis results are applicable to non-Asians and whether different ethnicity had different response to IBC. Large-scale clinical trials of IBC and NIBC to treat SCLC suggest that Asians and non-Asians can respond quite differently to chemotherapy. Whereas a clinical trial in Japanese patients (JCOG 9511) [21] reported better OS with irinotecan combined with platinum than with etoposide combined with platinum (12.8 vs 9.4mo., *p* = 0.002), a similar trial in North Americans (SWOG 0124) [7] reported similar OS for the two treatments (9.9 vs 9.1mo., *p* = 0.71). Moreover, efficacy of IBC to treat CRC differed significantly between Caucasians and African-American patients in a North American study (ORR: 41 % vs 28 %; *p* = 0.008) [22]. These evidences suggested that the ethnic bias may exist in the clinical response to IBC. However, this finding should be confirmed in larger cohorts of Asians and non-Asians with NSCLC, and it should inspire similar meta-analyses in cohorts with CRC or SCLC.

**Fig. 8:**
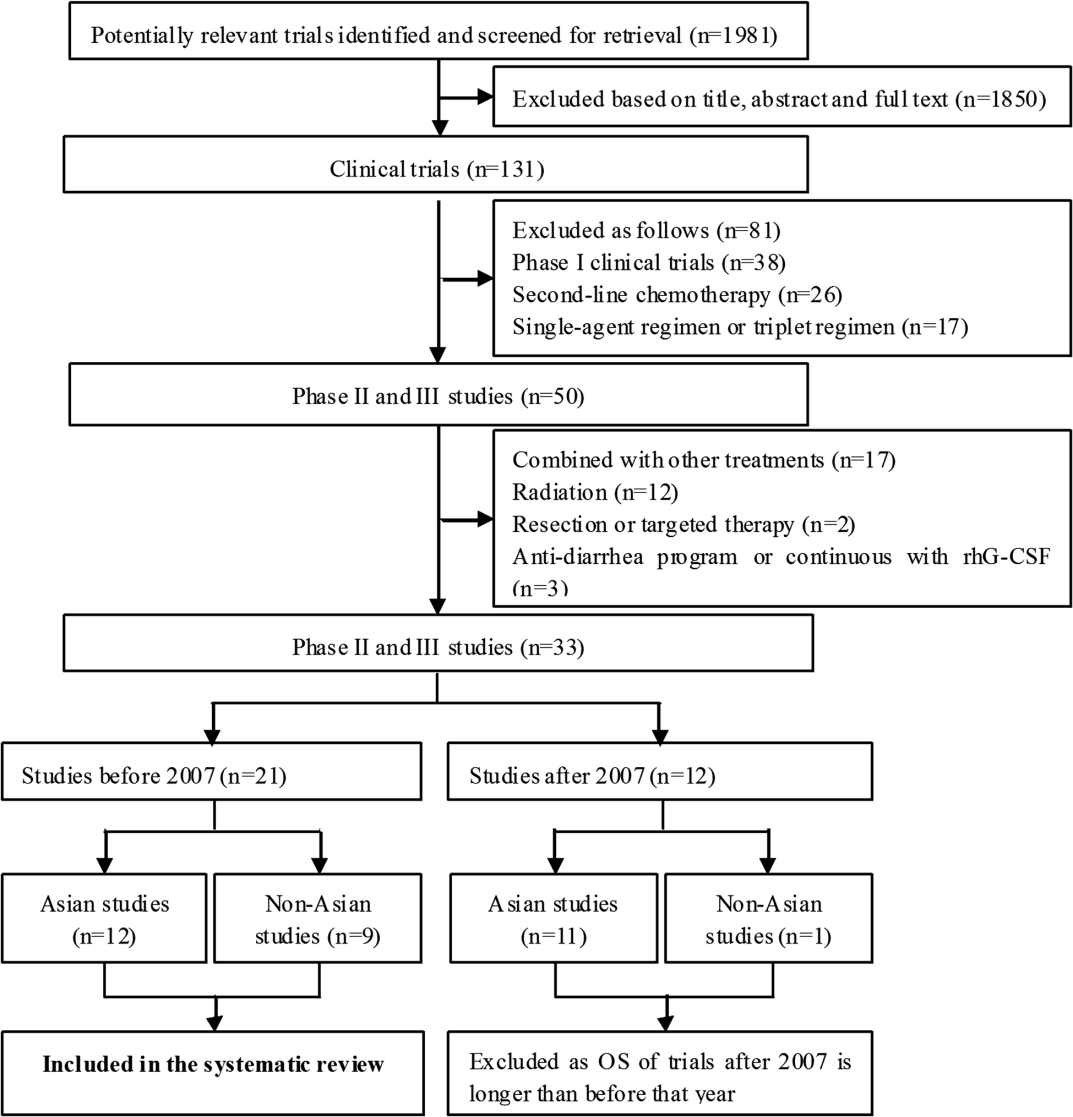
Trial identification and inclusion in the systematic review of irinotecan-based chemotherapy efficacy in different ethnicities: The literature was selected according to the criteria as follows: (1) NSCLC patients with previously untreated with chemotherapy; (2) treated with irinotecan-based doublet regimen; (3) prospective clinical trials Phase II and III

To further examine whether efficacy of IBC depends on patient ethnicity in an even larger cohort, we reviewed the literature for clinical trials of IBC to treat Asian and non-Asian patients with NSCLC, primarily in stage IIIB or IV. The search strategy and data extraction are consistent with the method used in the meta-analysis of this study. The selected criteria were as follows: (1) NSCLC patients with previously untreated with chemotherapy; (2) treated with irinotecan-based doublet regimen; (3) prospective clinical trials Phase II and III. We identified 33 Phase II and III clinical trials published from 1990 to 2014 (see Fig. 8 and Table 2). Even applying less strict inclusion criteria than those we used for the present meta-analysis, we found that 23 of the 33 trials involved Asian patients, while only 10 involved non-Asian patients. This ethnic imbalance was especially true among the 12 trials published after 2007, only one of which involved non-Asian patients. Since OS seemed substantially longer in trials published after 2007 than in trials before that year (14.5 vs 10.7mo. t = −4.518, *p* = 0.001), perhaps reflecting advances in targeted therapy, we excluded the 12 trials published after 2007. We performed statistical analysis on the remaining 21 trials, including 12 trials that involved 705 Asian patients [13, 14, 18, 23–31] and nine trials that involved 384 non-Asian patients, primarily Caucasians [19, 32–39]. Statistical Pearson Chi-Square or t-tests showed significantly higher ORR and a tendency toward longer OS in Asian patients than in non-Asian patients (ORR: 37.7 % vs 24.7 %, *χ*^2^ = 18,93, *p* < 0.001; OS: 11.2 vs 10.1mo., t = 2.036, *p* = 0.058). PFS, in contrast, was similar between the two populations (median 4.1 vs 4.7mo., t = −0.753, *p* = 0.467), probably as the data from Asian patients is too small and only five Asian trials reported the PFS data. Although these results did not come exclusively from RCTs or other controlled trials, their consistency with our meta-analysis suggests that IBC is significantly more effective for Asian patients with NSCLC than for non-Asian patients. Therefore, the results of this meta-analysis may not apply to non-Asian populations.

**Table 2:** Summary of phase II-III trials of Irinotecan-based doublet regimen on advanced NSCLC as first-line chemotherapy (2006–1990)*

Author	N	Races	Regimen	Efficacy			Toxicity	
				Median OS (month)	Median PFS (month)	RR (%)	Grade 3/ 4 Neut. (%)	Grade 3/4 Diarrea (%)
Asian trials								
Saito H et al.[23] 2006	27	Japanese	Irinotecan 60 mg/m^2^ day 1, 8, Cisplatin 60 mg/ m^2^ day 1, q 21 days	12.1	NR	30.0	60.0	22.0
Hino M et a[l24] 2006	39	Japanese	Irinotecan 60 mg/m^2^ on days 1, 8, 15, Cisplatin 30 mg/ m^2^ day 1, 8, 15, q 28 days	12.8	2.1	33.3	15.4	15.4
Zhang XR et al.[25] 2006	24	Chinese	Irinotecan 60 mg/m^2^ day 1, 8, 15, Cisplatin 80 mg/ m^2^ day 1, q 28 days	NR	NR	29.2	17.1	5.7
Fukuda M et al.[26] 2004	59	Japanese	Irinotecan 50 mg/m^2^ on days 1, 8 and 15, Carboplatin AUC 5, q 28 days	10.0	4.0	34.0	60.0	7.0
Yamamoto N et al.[14] 2004	57	Japanese	Irinotecan 60mg/m^2^ day 1, 8, Docetaxel 60mg/m^2^ day 8, q 21 days	10.7	4.2	32.0	84.0	16.0
Ichiki M et al.[27] 2003	44	Japanese	Irinotecan 80 mg/m^2^ day 1, 8, 15, Ifosfamide 1.5 g/m^2^ day 1 to 3, q 28 days	12.5	5.3	29.5	38.6	6.8
Negoro S et al.[13] 2003	133	Japanese	Irinotecan 60 mg/m^2^ day 1, 8, 15, Cisplatin 80 mg/m^2^ day 1, q 28 days	11.7	4.9	43.3	37.0 (grade 4)	12.0
Takeda K et al.[28] 2002	36	Japanese	Irinotecan 50 mg/m^2^ day 1, 8, 15, Carboplatin AUC 5 day 1, q 28 days	10.2	NR	25.0	76.5	5.9
Ueoka H et al.[30] 2001	44	Japanese	Irinotecan 50 mg/m^2^ day 1, 8, Cisplatin 60 mg/m^2^ day 1, 8, q 28days	12.5	NR	48	70.5	25
Nagao K et al.[29] 2000	69	Japanese	Irinotecan 65 mg/m^2^ day 1, 8, 15, Cisplatin 80 mg/m^2^ day 1, q 28 days	10.3	NR	47.8	80.3	18.8
Takiguchi Y et al.[18] 2000	104	Japanese	Irinotecan 60mg/m^2^ day 1, 8, 15, Cisplatin 80mg/m^2^ day 1, q 28 days	10.5	NR	29.0	50.0 (grade 4)	13.0
Masuda N et al.[31] 1998	69	Japanese	Irinotecan 60 mg/m^2^ day 1, 8, 15, Cisplatin 80 mg/m^2^ day 1, q 28 days	10.3	NR	52.0	80	19
Non-Asian trials								
Cardenal F et al.[37] 2003	73	Spanish	Irinotecan 200 mg/m^2^ day 1, Cisplatin 80 mg/m^2^ day 1, q 21 days	8.2	3.9	24.7	NR	29.0
Jagasia MH et al.[38] 2001	50	Americans	Irinotecan 65 mg/m^2^ and Cisplatin 30 mg/ m^2^ day 1, 8, 15, 21, q 42 days	11.6	6.9	36.0	26.0	26.0
DeVore RF et al.[39] 1999	52	Americans	Irinotecan 60 mg/m^2^ day 1, 8, 15, Cisplatin 80 mg/m^2^ day 1, q 28 days	9.9	5.1	28.8	46.1	17.3
Pillot GA et al.[32] 2006	42	Americans	Irinotecan 200 mg/m^2^ day 1 , Carboplatin AUC 5 day 1, q 21days	11.7	6.9	14.0	62.0	5.0
Ziotopoulos P et al.[33] 2005	39	Greeks	Irinotecan 200 mg/m^2^ day 1, Docetaxel 80 mg/m^2^ day 1, q 21 days	10.8	3.0	23.0	28.2	23.1
Stathopoulos GP et al.[34] 2005	52	Greeks	Irinotecan 125 mg/m^2^ day 1, 8, Paclitaxel 135 mg/m^2^ day 1, q 21 days	NR	6.0	41.0	NR	NR
Murren JR et al.[35] 2005	23	Americans	Irinotecan 50 mg/m^2^ and Paclitaxel 75 mg/m^2^ on days 1 and 8 , q 21 days	9.2	2.8	9.0	26.0	5.0
Raez LE et al.[36] 2004	14	Irish	Irinotecan 50mg/m^2^ day 1, 8, 15, Docetaxel 50mg/m^2^ day 2, q 28 days	11.0	NR	7.0	21.0	0
Rocha Lima CM et al.[19] 2004	39	Americans	Irinotecan 100 mg/ m^2^ day 1, Gemcitabine 1000 mg/ m^2^ day 1, 8, q 21 days	8.0	3.5	12.8	26.0	13.0

*NR* not reported, *Ad*. adenocarcinoma, *Neut*. neutropenia

*The literature was selected according to the criteria as follows: (1) NSCLC patients with previously untreated with chemotherapy; (2) treated with irinotecan-based doublet regimen; (3) prospective clinical trials Phase II and III.

Then what causes efficacy difference in populations? Actually, except that the clinical response to IBC is dependent on the ethnic in NSCLC, the clinical response to other drugs in NSCLC is also different with different population. For example, it has been proved that bevacizumab in combination with chemotherapy can improve clinical outcomes in patients with advanced or recurrent NSCLC. However, compared with global data from SAiL study (ORR 50.7 %, median TTP 7.8mo., median OS 14.6mo.) [40], Asian patients, especially Chinese population, with advanced non-squamous NSCLC seemed to have an advantage over other ethnicities on overall survival after receiving bevacizumab-based first-line treatment (ORR 68.9 %, median TTP 8.8mo., median OS 18.5mo.) [41]. Also, as the rate of EGFR positive mutation is more than 30 % in Asian patients and less than 10 % in Caucasians [4], EGFR-TKI treating NSCLC had higher response in Asian patients than in Caucasians. Then we suspected that different genotype may account for the different clinical response to IBC in different populations. As we know, the SNP of UGT1A1 gene is the hotspot of research at present. Frequencies of these variants depend on ethnicity, with UGT1A1*28 being found more commonly in Caucasians and a higher frequency of UGT1A1*6 being found among Asians [42, 43], both of which were associated with irinotecan-induced severe neutropenia and diarrhea. A study from Korean patients with NSCLC administered irinotecan found that the SNP type of UGT1A1*6 related to the response rate and survival [44]. Thus further study must be investigated into the relationship of SNP of UGT1A1 gene or other genes with the response on NSCLC in different populations.

Our meta-analysis aggregated patients with various histological types of advanced NSCLC, but there is evidence to suggest that irinotecan efficacy may depend on histological type. In one meta-analysis, nedaplatin + irinotecan was more active against squamous cell carcinoma than against non-squamous cell carcinoma, based on overall response rate (51.9 vs 35.1 %, *p* = 0.115) and OS (14.5 vs 9.1mo., *p* = 0.127) [45]. Future studies should examine how IBC efficacy varies with histological type of NSCLC, preferably in different ethnicities. We suggest that, regardless of the outcomes of these studies, IBC may be particularly appropriate for treating squamous cell lung carcinoma, given the scarcity of targeted therapies for this carcinoma and a median survival of only 8–10 months, overall response rates of 25 %–35 % with platinum-based therapy [46]. However, clinicians should be aware that the risk of grade 3–4 vomiting and diarrhea is higher with IBC than NIBC, based on our meta-analysis.

## Conclusions

In summary, we have found that the efficacy of an irinotecan-based doublet chemotherapy regimen against advanced NSCLC is similar to that of a non-irinotecan-based doublet regimen, at least in Asian patients. Therefore we recommend IBC as first-line chemotherapy in Asian patients, especially for squamous cell lung carcinoma and the patients being not suitable for target therapy. Our results should be interpreted with caution because some trials in our meta-analysis used non-platinum regimens, while the gold standard for treating NSCLC is a platinum-based doublet regimen. Also, the ethnicity for irinotecan seems to be essential and a mixed investigated population may dilute the conclusions. Future meta-analyses should be conducted to compare platinum + irinotecan with platinum + other agents. Also,as EGFR mutant status related to the efficacy of chemotherapy and non-Asian patients have different mutant status with Asian patients, investigation of the activity of irinotecan-based regimens in EGFR mutant patients should also be urged.

## Additional file

Additional file 1:
**Efficacy and toxicity of the seven trials.**  (DOC 64 kb)

## Abbreviations

IBC: Irinotecan-based chemotherapy
NIBC: Non-irinotecan-based chemotherapy

## References

[1] Bulzebruck H, Bopp R, Drings P, Bauer E, Krysa S, Probst G, van Kaick G, Muller KM, Vogt-Moykopf I (1992). New aspects in the staging of lung cancer. Prospective validation of the international union against cancer TNM classification. Cancer.

[2] Yang P, Allen MS, Aubry MC, Wampfler JA, Marks RS, Edell ES, Thibodeau S, Adjei AA, Jett J, Deschamps C (2005). Clinical features of 5,628 primary lung cancer patients: experience at Mayo Clinic from 1997 to 2003. Chest.

[3] Govindan R, Page N, Morgensztern D, Read W, Tierney R, Vlahiotis A, Spitznagel EL, Piccirillo J (2006). Changing epidemiology of small-cell lung cancer in the United States over the last 30 years: analysis of the surveillance, epidemiologic, and end results database. J Clin Oncol.

[4] Boolell V, Alamgeer M, Watkins DN, Ganju V (2015). The evolution of therapies in Non-small cell lung cancer. Cancers.

[5] National Comprehensive Cancer Network. Non-small cell lung cancer v.3. 2015. http://www.nccn.org/professionals/physician_gls/f_guidelines.asp 19817021

[6] Rothenberg ML, Eckardt JR, Kuhn JG, Burris HA, Nelson J, Hilsenbeck SG, Rodriguez GI, Thurman AM, Smith LS, Eckhardt SG (1996). Phase II trial of irinotecan in patients with progressive or rapidly recurrent colorectal cancer. J Clin Oncol.

[7] Lara PN, Natale R, Crowley J, Lenz HJ, Redman MW, Carleton JE, Jett J, Langer CJ, Kuebler JP, Dakhil SR (2009). Phase III trial of irinotecan/cisplatin compared with etoposide/cisplatin in extensive-stage small-cell lung cancer: clinical and pharmacogenomic results from SWOG S0124. J Clin Oncol.

[8] Lim WT, Lim ST, Wong NS, Koo WH (2003). CPT-11 and cisplatin in the treatment of advanced gastric cancer in Asians. J Chemother.

[9] Yamamoto K, Kokawa K, Umesaki N, Nishimura R, Hasegawa K, Konishi I, Saji F, Nishida M, Noguchi H, Takizawa K (2009). Phase I study of combination chemotherapy with irinotecan hydrochloride and nedaplatin for cervical squamous cell carcinoma: Japanese gynecologic oncology group study. Oncol Rep.

[10] Tierney JF, Stewart LA, Ghersi D, Burdett S, Sydes MR (2007). Practical methods for incorporating summary time-to-event data into meta-analysis. Trials.

[11] Banares R, Albillos A, Rincon D, Alonso S, Gonzalez M, Ruiz-del-Arbol L, Salcedo M, Molinero LM (2002). Endoscopic treatment versus endoscopic plus pharmacologic treatment for acute variceal bleeding: a meta-analysis. Hepatology.

[12] Higgins JP, Thompson SG, Deeks JJ, Altman DG (2003). Measuring inconsistency in meta-analyses. BMJ.

[13] Negoro S, Masuda N, Takada Y, Sugiura T, Kudoh S, Katakami N, Ariyoshi Y, Ohashi Y, Niitani H, Fukuoka M (2003). Randomised phase III trial of irinotecan combined with cisplatin for advanced non-small-cell lung cancer. Br J Cancer.

[14] Yamamoto N, Fukuoka M, Negoro SI, Nakagawa K, Saito H, Matsui K, Kawahara M, Senba H, Takada Y, Kudoh S (2004). Randomised phase II study of docetaxel/cisplatin vs docetaxel/irinotecan in advanced non-small-cell lung cancer: a west Japan thoracic oncology group study (WJTOG9803). Br J Cancer.

[15] Ohe Y, Ohashi Y, Kubota K, Tamura T, Nakagawa K, Negoro S, Nishiwaki Y, Saijo N, Ariyoshi Y, Fukuoka M (2007). Randomized phase III study of cisplatin plus irinotecan versus carboplatin plus paclitaxel, cisplatin plus gemcitabine, and cisplatin plus vinorelbine for advanced non-small-cell lung cancer: four-Arm cooperative study in Japan. Ann Oncol.

[16] Han JY, Lee DH, Song JE, Lee SY, Kim HY, Kim HT, Lee JS (2008). Randomized phase 2 study of irinotecan plus cisplatin versus gemcitabine plus vinorelbine as first-line chemotherapy with second-line crossover in patients with advanced nonsmall cell lung cancer. Cancer.

[17] Zhao WY, Chen DY (2012). [A prospective randomized controlled clinical trial of irinotecan plus cisplatin versus gemcitabine plus cisplatin as a first-line treatment for advanced non-small cell lung cancer]. Zhonghua zhong liu za zhi [Chinese J Oncol.

[18] Takiguchi Y, Nagao K, Nishiwaki Y, Yokoyama A, Saijo N, Ohashi Y, Niitani H (2000). The final results of a randomized phase III trial comparing irinotecan (CPT-11) and cisplatin (CDDP) with vindesine (VDS) and CDDP in advanced non-small cell lung cancer (NSCLC). Lung Cancer (Amsterdam, Netherlands).

[19] Rocha Lima CM, Rizvi NA, Zhang C, Herndon JE, Crawford J, Govindan R, King GW, Green MR, Cancer Leukemia Group B (2004). Randomized phase II trial of gemcitabine plus irinotecan or docetaxel in stage IIIB or stage IV NSCLC. Ann Oncol.

[20] de Forni M, Bugat R, Chabot GG, Culine S, Extra JM, Gouyette A, Madelaine I, Marty ME, Mathieu-Boue A (1994). Phase I and pharmacokinetic study of the camptothecin derivative irinotecan, administered on a weekly schedule in cancer patients. Cancer Res.

[21] Noda K, Nishiwaki Y, Kawahara M, Negoro S, Sugiura T, Yokoyama A, Fukuoka M, Mori K, Watanabe K, Tamura T (2002). Irinotecan plus cisplatin compared with etoposide plus cisplatin for extensive small-cell lung cancer. N Engl J Med.

[22] Sanoff HK, Sargent DJ, Green EM, McLeod HL, Goldberg RM (2009). Racial differences in advanced colorectal cancer outcomes and pharmacogenetics: a subgroup analysis of a large randomized clinical trial. J Clin Oncol.

[23] Saito H, Kudoh S, Nakagawa K, Negoro S, Matsui K, Semba H, Takada M (2006). Phase II study of 3-week scheduling of irinotecan in combination with cisplatin in patients with advanced nonsmall-cell lung cancer. Am J Clin Oncol.

[24] Hino M, Kobayashi K, Yoshimura A, Takeda Y, Hisakatsu S, Yoneda S, Gemma A, Moriya H, Kudoh S, East Japan Chesters G (2006). Weekly administration of irinotecan (CPT-11) plus cisplatin for non-small cell lung cancer. Anticancer Res.

[25] Zhang XR, Zhu YZ, Xiu QY, Han FC, Liu DQ, Chu DT (2006). [Irinotecan plus cisplatin for the treatment of advanced non-small cell lung cancer]. Zhonghua zhong liu za zhi [Chinese J Oncol].

[26] Fukuda M, Oka M, Soda H, Kinoshita A, Fukuda M, Nagashima S, Kuba M, Takatani H, Tsurutani J, Nakamura Y (2004). Phase II study of irinotecan combined with carboplatin in previously untreated non-small-cell lung cancer. Cancer Chemother Pharmacol.

[27] Ichiki M, Rikimaru T, Gohara R, Koga T, Kawayama T, Matunami M, Oshita Y, Kamimura T, Aizawa H (2003). Phase II study of irinotecan and ifosfamide in patients with advanced non-small cell lung cancer. Oncology.

[28] Takeda K, Takifuji N, Uejima H, Yoshimura N, Terakawa K, Negoro S (2002). Phase II study of irinotecan and carboplatin for advanced non-small cell lung cancer. Lung Cancer.

[29] Nagao K, Fukuoka M, Fujita A, Kurita Y, Saito R, Niitani H, Negoro S, Katakami N, Nakano M (2000). A phase II study of irinotecan combined with cisplatin in non-small cell lung cancer. CPT-11 lung cancer study group. Gan Kagaku ryoho Cancer Chemotherapy.

[30] Ueoka H, Tanimoto M, Kiura K, Tabata M, Takigawa N, Segawa Y, Takata I, Eguchi K, Okimoto N, Harita S (2001). Fractionated administration of irinotecan and cisplatin for treatment of non-small-cell lung cancer: a phase II study of Okayama lung cancer study group. Br J Cancer.

[31] Masuda N, Fukuoka M, Fujita A, Kurita Y, Tsuchiya S, Nagao K, Negoro S, Nishikawa H, Katakami N, Nakagawa K (1998). A phase II trial of combination of CPT-11 and cisplatin for advanced non-small-cell lung cancer. CPT-11 lung cancer study group. Br J Cancer.

[32] Pillot GA, Read WL, Hennenfent KL, Marsh S, Gao F, Viswanathan A, Cummings K, McLeod HL, Govindan R (2006). A phase II study of irinotecan and carboplatin in advanced non-small cell lung cancer with pharmacogenomic analysis: final report. J Thoracic Oncol.

[33] Ziotopoulos P, Androulakis N, Mylonaki E, Chandrinos V, Zachariadis E, Boukovinas I, Agelidou A, Kentepozidis N, Ignatiadis M, Vossos A (2005). Front-line treatment of advanced non-small cell lung cancer with irinotecan and docetaxel: a multicentre phase II study. Lung Cancer.

[34] Stathopoulos GP, Dimitroulis J, Antoniou D, Katis C, Tsavdaridis D, Armenaki O, Marosis C, Michalopoulou P, Grigoratou T, Stathopoulos J (2005). Front-line paclitaxel and irinotecan combination chemotherapy in advanced non-small-cell lung cancer: a phase I-II trial. Br J Cancer.

[35] Murren JR, Andersen N, Psyrri D, Brandt D, Nadkarni R, Rose M, Davies MJ, Parisot N, Rosenfield AT, Pizzorno G (2005). Evaluation of irinotecan plus paclitaxel in patients with advanced non-small cell lung cancer. Cancer Biol Therapy.

[36] Raez LE, Rosado MF, Santos ES, Reis IM (2004). Irinotecan and docetaxel as first line chemotherapy in patients with stage IIIB/IV non-small cell lung cancer--experience from a prematurely closed phase II study. Lung Cancer.

[37] Cardenal F, Domine M, Massuti B, Carrato A, Felip E, Garrido P, Juan O, Artal A, Barneto I, Lopez-Vivanco G (2003). Three-week schedule of irinotecan and cisplatin in advanced non-small cell lung cancer: a multicentre phase II study. Lung Cancer.

[38] Jagasia MH, Langer CJ, Johnson DH, Yunus F, Rodgers JS, Schlabach LL, Cohen AG, Shyr Y, Carbone DP, Devore RF (2001). Weekly irinotecan and cisplatin in advanced non-small cell lung cancer: a multicenter phase II study. Clin Cancer Res.

[39] DeVore RF, Johnson DH, Crawford J, Garst J, Dimery IW, Eckardt J, Eckhardt SG, Elfring GL, Schaaf LJ, Hanover CK (1999). Phase II study of irinotecan plus cisplatin in patients with advanced non-small-cell lung cancer. J Clin Oncol.

[40] Crino L, Dansin E, Garrido P, Griesinger F, Laskin J, Pavlakis N, Stroiakovski D, Thatcher N, Tsai CM, Wu YL (2010). Safety and efficacy of first-line bevacizumab-based therapy in advanced non-squamous non-small-cell lung cancer (SAiL, MO19390): a phase 4 study. Lancet Oncol.

[41] Zhou CC, Bai CX, Guan ZZ, Jiang GL, Shi YK, Wang MZ, Wu YL, Zhang YP, Zhu YZ (2014). Safety and efficacy of first-line bevacizumab combination therapy in Chinese population with advanced non-squamous NSCLC: data of subgroup analyses from MO19390 (SAiL) study. Clin Translation Oncol.

[42] Liu CY, Chen PM, Chiou TJ, Liu JH, Lin JK, Lin TC, Chen WS, Jiang JK, Wang HS, Wang WS (2008). UGT1A1*28 polymorphism predicts irinotecan-induced severe toxicities without affecting treatment outcome and survival in patients with metastatic colorectal carcinoma. Cancer.

[43] Park SR, Kong SY, Rhee J, Park YI, Ryu KW, Lee JH, Kim YW, Choi IJ, Kim CG, Lee JY (2011). Phase II study of a triplet regimen of S-1 combined with irinotecan and oxaliplatin in patients with metastatic gastric cancer: clinical and pharmacogenetic results. Annals of Oncol.

[44] Han JY, Lim HS, Shin ES, Yoo YK, Park YH, Lee JE, Jang IJ, Lee DH, Lee JS (2006). Comprehensive analysis of UGT1A polymorphisms predictive for pharmacokinetics and treatment outcome in patients with non-small-cell lung cancer treated with irinotecan and cisplatin. J Clin Oncol.

[45] Oshita F, Honda T, Murakami S, Kondo T, Saito H, Noda K, Yamada K (2011). Comparison of nedaplatin and irinotecan for patients with squamous and nonsquamous cell carcinoma of the lung: meta-analysis of four trials. J Thoracic Oncol.

[46] Schiller JH, Harrington D, Belani CP, Langer C, Sandler A, Krook J, Zhu J, Johnson DH, Eastern Cooperative Oncology G (2002). Comparison of four chemotherapy regimens for advanced non-small-cell lung cancer. N Engl J Med.

